# Application of traditional Chinese medicine in film drug delivery system

**DOI:** 10.3389/fphar.2022.956264

**Published:** 2022-10-10

**Authors:** Qianhang Li, Feng Luo, Pingnan Jiang, Chenxi Feng, Feifei He, Lina Dong, Delin Xu, Junhua Shi

**Affiliations:** ^1^ Department of Radiology, Affiliated Hospital of Zunyi Medical University, Zunyi, China; ^2^ Department of Cell Biology, Zunyi Medical University, Zunyi, Guizhou, China

**Keywords:** traditional Chinese medicine (TCM), film agent, preparation processes, clinical application, drug delivery systems

## Abstract

Film drug delivery systems have the advantages of precise administration, simple process and easy portability, compared with other traditional drug delivery systems such as tablets, capsules, syrups, ointments, etc. The traditional Chinese medicine (TCM) are normally developed in four categories of film agent like patch film, coating, spray film and gel film, which are applied to the treatment of oral ulcers, chronic diseases of lower limbs, burns, scalds, gynecological disease and body care. So the TCM film has great research value and prominent market prospect. In this review, we summarized the research progress of the material composition, pharmaceutical production, clinical application and pharmacology mechanism of various TCM film agents. It may provide a comprehensive reference for further development and utilization of TCM film agents.

## 1 Introduction

Ethnic Chinese medicinal materials have thousands of years of history in clinical application, while how to achieve accurate drug delivery and precision pharmaceutical care has becoming an urgent scientific problem to be solved. Films are preparations made by mixing drugs and film-forming materials evenly, which films can be pasted and fixed in one place, which can produce local or systemic therapeutic effects. Based on the former published paper, film agent has being used in clinically related diseases of stomatology, otolaryngology, ophthalmology, gynecology, dermatology and other departments. The TCM film agent has the advantages of simple and scientific preparation processes, high curative effect, small dose and small volume, lightweight and so on.

At present, with the maturity of nanomaterial research and development technology, various high-tech technologies such as 3D printing and liquid crystal technology are based on nanomaterials and are applied to the development of film formulations (Tong et al., 2018; Hao et al., 2019). It is hoped that more precise targeted therapy can be achieved and the process of intelligent drug research and development can be promoted. At the same time, in order to more accurately evaluate the overall quality of the film, the continuous introduction of new test methods can fully reflect the effect of each link in the preparation process on the final film, thereby helping the R&D personnel to further improve the process. In addition, the latest research direction of TCM film concentrates on the elucidation of the effective mechanism of TCM for disease treatment, which mainly starts from the regulation of related genes, the expression of related proteins, enzymes and factors, and the impact of related signaling pathways (Huang and Wang., 2022).

We learned from the China National Drug Administration and Monitoring Bureau that there are many traditional Chinese medicine films on the market. The common ones are patch film, film coating, and spray film, and there are fewer spray film agents. According to statistics, there are few TCM films listed abroad, and most of them are developed and produced in China. We have listed some typical listed TCM films and their indications (Table 1).

**TABLE 1:** The number of Chinese medicine film preparations in the article and their main uses.

Type	Name of film agent	Indications	R and D enterprise
Film coating agent	Acne film coating	Clear heat and dry dampness, cool blood and detoxify, remove blood stasis and disperse knots. For the adjuvant treatment of acne vulgaris with accumulation of damp-heat and blood-heat stasis	Beijing Huashen Pharmaceutical Co., Ltd.
Film coating agent	Shutongan coating agent	Relaxing tendons and promoting blood circulation, reducing swelling and relieving pain	Hebei Jinzhong Pharmaceutical Co., Ltd.
Film coating agent	Snow Mountain Golden Luohan Pain Relief Film	Promote blood circulation, reduce swelling, relieve pain. For acute and chronic sprains, rheumatoid arthritis, rheumatoid arthritis, gout, frozen shoulder, pain and swelling of limbs and joints caused by bone hyperplasia, and neuropathic headaches	Tibet Nuodikang Pharmaceutical Co., Ltd.
Film coating agent	Danxiong scar film	Promote blood circulation, soften firmness, relieve itching	Weihai Life Pharmaceutical Co., Ltd.
Film coating agent	Sodium fluoride anti-caries coating agent	For the prevention of caries	Shenzhen Zhongke Jingcheng Medical Technology Co., Ltd.
Patch film agent	Sophora film	Clear heat and dry dampness, cool blood and detoxify, remove blood stasis and disperse knots. For the adjuvant treatment of acne vulgaris with accumulation of damp-heat and blood-heat stasis	Guizhou Dexuantang Hukang Pharmaceutical Co., Ltd.
Patch film agent	Boxingkang medicine film	Clearing away heat and detoxifying, drying dampness and killing insects, dispelling wind and relieving itching. For vaginal diseases (Trichomonas vaginitis, fungal vaginitis, acute and chronic cervicitis)	Guizhou Shengjitang Pharmaceutical Co., Ltd.
Patch film agent	Propolis oral film	Clearing heat and relieving pain. For recurrent aphthous ulcers.	China Resources Zizhu Pharmaceutical Co., Ltd.
Patch film agent	Erxiekang film	Warm in the middle to dispel cold and stop diarrhea. For non-infectious diarrhea in children	Shanxi Jinxin Shuanghe Pharmaceutical Co., Ltd.
Patch film agent	Chuanhua Pain Relief Film	Promote blood circulation to remove blood stasis, dispel cold and relieve pain. For rheumatism pain, bruise pain, bone hyperplasia, cervical spondylosis, frozen shoulder, lumbar muscle strain and other pain.	Jilin Province Huinan Changlong Biochemical Pharmaceutical Co., Ltd.
Gel film agent	Chitosan film former	Apply to cervical ulcer to promote ulcer healing	Heilongjiang Yunjia Medical Technology Co., Ltd.
Gel film agent	Tsubaki Gel	Clear heat and dry dampness, remove blood stasis and promote muscle	Zhuzhou Qianjin Pharmaceutical Co., Ltd.
Gel film agent	Sore Gel	For reducing swelling and analgesia, promoting blood circulation and removing blood stasis, relaxing tendons and collaterals, dissolving sputum and dissipating knots, bruises, rheumatism and joint pain, frozen shoulder, gout, and hyperplasia of breast lobules	Yunnan Baiyao Group Co., Ltd.
Gel film agent	Kangfu Gel	Expelling wind and dampness, relieving itching and killing insects, antiseptic and muscle regeneration. Used for vulvar or vaginal congestion, swelling, burning, pain, increased secretions or local ulcers, erosions, itching, etc. caused by vulvitis, vulvar ulcers, vaginitis, etc	Guizhou Jianxing Pharmaceutical Co., Ltd.
Gel film agent	Baofukang Gel	For vaginal discharge caused by damp-heat stasis, see vaginal pruritus, fungal vaginitis, senile vaginitis, cervical erosion when the amount of vaginal discharge is multi-colored and yellow	Sinopharm Group Zhonglian Pharmaceutical Co., Ltd.
Spray film agent	Biological wound spray	This product is suitable for the cleaning and care of superficial wounds in the superficial dermis and above, and has the functions of hemostasis, antibacterial and wound protection	Changsha Ruitai Medical Technology Co., Ltd.

**FIGURE 1:**
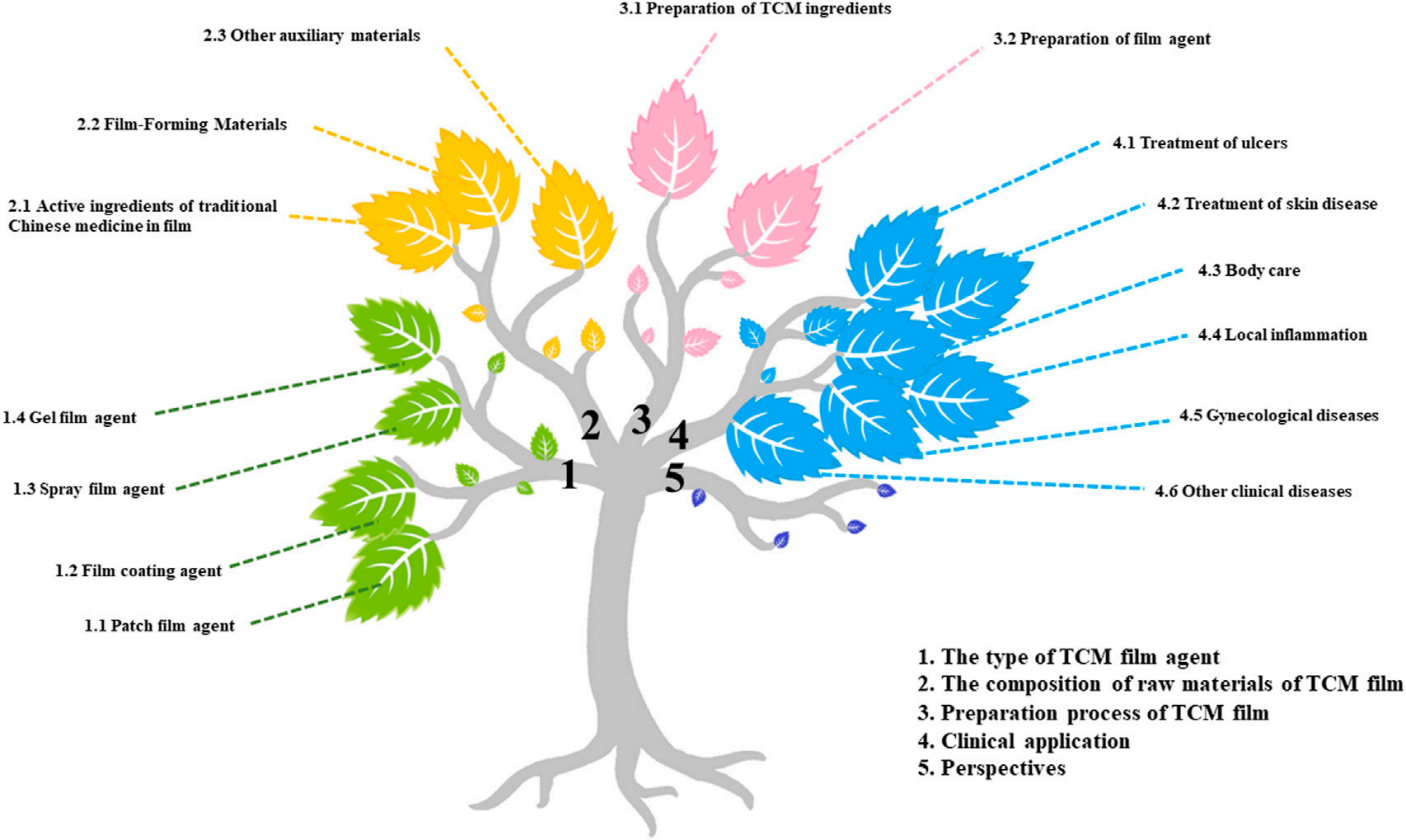
Mind map of the article.

This paper summarizes the pharmaceutical process and clinical application of TCM films in recent years, and puts forward ideas and prospects. It may provide a clue for the following development and utilization of TCM film and the expansion and upgrading of clinical applications (Figure 1).

## 2 The type of TCM film agent

There are many kinds of TCM film agents and their classification methods are also different. In the light of the structure, they can be classified into monolayer film, double-layer film and sandwich film. According to different application sites can be roughly categorized into oral film, eye film, nasal film, vaginal film, skin or mucosal external film, periodontal film and implant film. Film preparations are roughly divided into three categories: liquid preparations, semi-solid preparations, and solid preparations according to their physical form, and their use methods are different. Liquid preparations include spray film, and the method of use is spray film formation. Semi-solid preparations include film coating agent and gel film agent, and the method of use is smearing to form a film. Solid preparations are applied directly to the affected area. Due to the different use methods can be roughly divided into patch film, coating film, spray film and gel film (Kathe and Kathpalia., 2017)**.** This paper summarizes the classification and application of the following four common film agents.

### 2.1 Patch film agent

**FIGURE 2:**
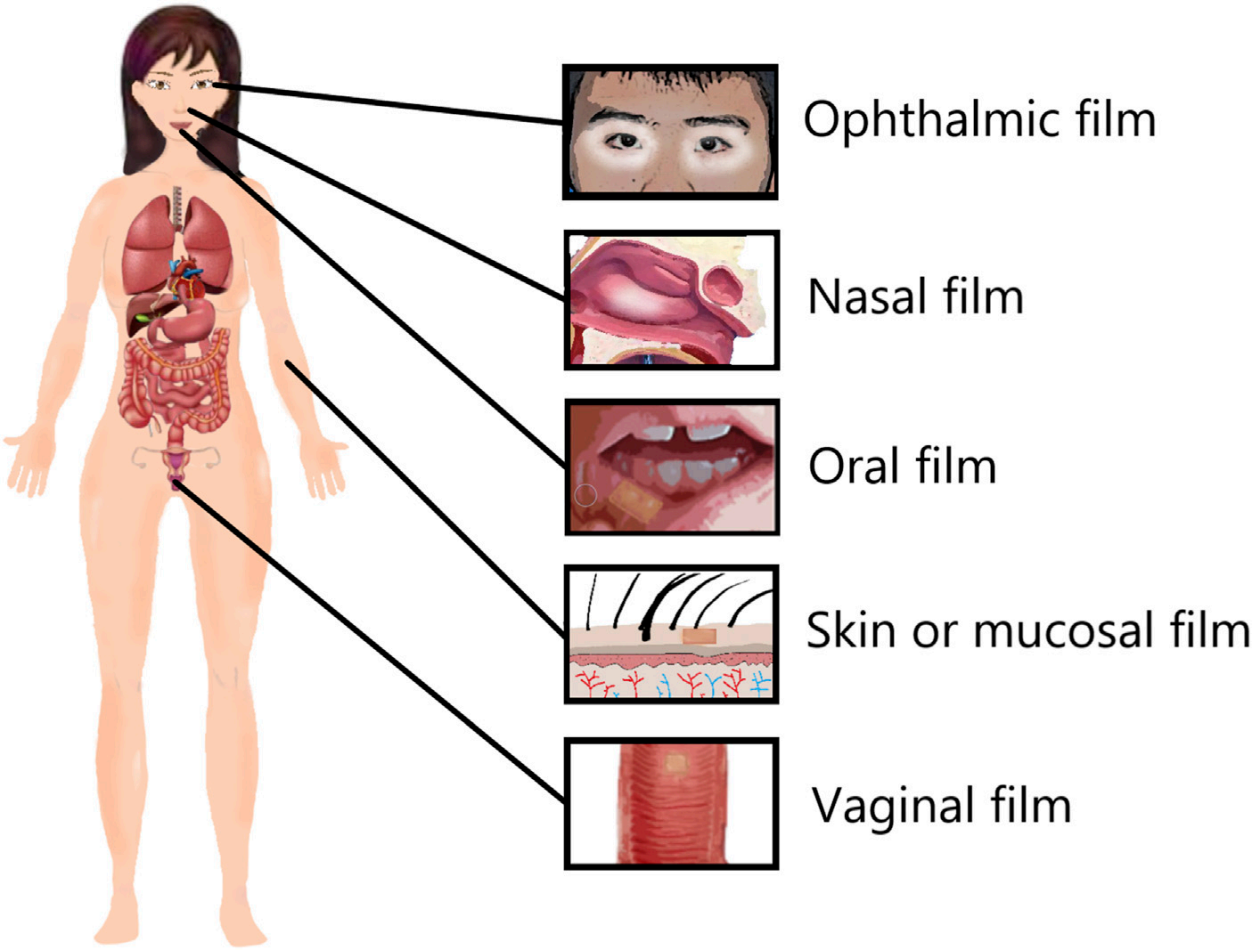
Classification of patch film agent.

Patch film agent refers to some TCM mixed with film-forming materials made of convenient and portable lamellar film. Because of its sustainable adhesion in the specific surface of a wound, it can be used for the treatment of the diseases in the oral cavity, eyes, nasal cavity or vagina, through the skin and mucous membrane percutaneous treatment of specific parts of the disease (Figure 2). The self-made Kouyanqing sustained-release film made of Kouyanqing granules and polyvinyl alcohol by Li et al. has a good clinical effect on oral ulcers and can relieve the pain symptoms of patients (Li et al., 2013). Yuan et al. showed that the film made of compound broad-leaf valerian, safflower, camphor wood, impatiens and carbomer had strong analgesic and anti-inflammatory activities (Yuan et al., 2013). Studies have shown that the patch film can also be used to treat lumbar muscle strain. Li et al. used modern technology to make seven kinds of traditional Chinese medicines such as angelica, safflower, and angelica into dry powder paste, and added the volatile oil and borneol extracted by distillation into ethanol solution to make a diluent, and combined the two to make a patch. When applied to the waist, the drug can quickly reach the diseased part, making the local meridians unobstructed, promoting blood circulation and removing blood stasis, dispelling wind and dispelling cold, and disappearing symptoms such as low back pain (Li et al., 2005). In addition, some studies have shown that the traditional Chinese medicine film can also be used to treat angina pectoris. Among the 35 cases in the traditional Chinese medicine film treatment group, 24 cases were cured, and the recovery rate accounted for 68.57%. The role of myocardial ischemia is a safe and effective traditional Chinese medicine preparation that can be used for the treatment of coronary heart disease and angina pectoris (Niu et al., 2007). All in all, the patch film has the advantages of being convenient to carry, simple to use, and simple to prepare. It is widely used in several clinical fields and is one of the most popular film formulations.

### 2.2 Film coating agent

Film coating agent refers to a liquid preparation in which some Chinese medicines are dissolved or dispersed in the solvent containing film-forming materials, and the solvent volatilizes after applying the affected area to form a film. It can be used for the treatment of skin burns, wound infection, acne, rash, eczema, surface anesthesia, soft tissue injury, etc. The compound membrane (CPCF) made by curcumin, PVA and collagen can effectively promote the healing of skin wounds. The experiment showed that the film had obvious inhibition zone to *Staphylococcus aureus* and *Escherichia coli,* and the wound healing rate of rats 15 days after operation was 98.03 ± 0.79%, which were higher than those of the control group (Leng et al., 2020). Wang et al. made the film coating agent with nine TCMs, which is stable in administration and has a good therapeutic effect on skin scars. The total cure rate was 88.69%, which was significantly higher than that of the control group (6.48%), and the inhibition rate of the drug on the proliferation and growth of fibroblasts detected by the MTT method was 83.27 ± 7.33% (Wang et al., 2018). It is also shown that the coating agent can also be applied to the field of frostbite prevention. Nanogel coating agent prepared from *Ganoderma lucidum* (GLT) containing triterpenoids has an obvious therapeutic effect on frostbite in rats. Among them, the combination of GLT nanogel and TUS has a beneficial effect on the healing process of frostbite by increasing the survival area and improving the pathological tissue in frostbite rats (Shen et al., 2016). In addition, clinical application shows that film coating agent can also nourish and whiten the skin. The surface coating agent is made of white *Poria cocos*, Trichosanthin, *Bletilla striata* and other TCMs, which can clear away heat, remove dampness and detoxify the skin with the help of the active ingredients of the medicine, and has a significant therapeutic effect on chloasma (Chen et al., 2018). In short, the coating agent is widely used in the clinical field because of its simple preparation, convenient use and significant curative effect.

### 2.3 Spray film agent

Spray film agent refers to some TCMs and suitable auxiliary materials filled in containers, released in mist when used. It could be directly sprayed to the skin and mucous membranes to form a film for treating the skin and mucous membrane and other parts of the disease. Former researchers found that the spray film made of *Rhizoma bolbostemmae*, PVP and carbomer has antiviral and antibacterial effects, which could be used to treat infectious skin diseases, and has a significant clinical effect in the treatment of condyloma acuminatum (Ye et al., 2013). In addition, clinical applications have shown that spray film can treat limb swelling, bruises, etc. For example, 36 cases of traumatic limb swelling were successfully treated with *Xiaoding* spray film (Xin, 2012). Some studies have also shown that the spray film of removing blood stasis and swelling made of peach seed, Chuanxiong rhizome, *Angelica* root, PVP-k30 and PVA 1788. This film has the effects of promoting blood circulation, removing blood stasis, swelling and relieving pain, mainly used for soft tissue swelling, ecchymosis, stasis and heat in the early stage of injury (Zhu et al., 2015). In general, spray film agents can not only protect the wound surface but also release drugs to play a local or systemic therapeutic effect, which is expected to provide a direction for the clinical treatment of skin mucosal injury and percutaneous treatment.

### 2.4 Gel film agent

Gel film is a thick liquid or semi-solid preparation with gel properties, being made by certain drugs and excipients that can form a gel or a thin film on the affected area. In most clinical applications, the use of dressings that maintain a moist environment promotes proper wound healing, such as hydrogels have good fluid absorption capacity, water retention capacity, water vapor transmission rate and integrity value. The hydrogel has an immediate response to gel formation when in contact with wound exudate, and high fluid absorption occurs through strongly hydrophilic gel formation, with potential use in biomedical applications (Jantrawut et al., 2019)**.** Summarizing dressings for superficial and partial thickness burns, Wasiak et al. found that hydrogels have a greater ability to absorb fluids and therefore can cope with higher levels of wound exudate, and their fluid-donating properties may also help wounds debridement and helps maintain a moist wound environment. In addition, burn wounds covered with hydrogel dressings healed faster than those treated with various conventional care regimens (Wasiak et al., 2013)**.** Lu et al. reviewed four commonly used methods for preparing soluble chemical cross-linked hydrogels, environmentally sensitive physical cross-linked hydrogels and supramolecular self-assembled hydrogels, providing ideas for designing traditional Chinese medicine gel film agents for clinical use (Lu et al., 2018). Studies have shown that sodium alginate (SA) and pectin (PC) as film-forming materials, crosslinking Ca^2+^ leads to the formation of a strong hydrogel, which can absorb a large amount of water, but is insoluble in aqueous solution and has enhanced mechanical properties and fluid absorption capacity, which is expected to be applied in the field of TCM and guide the direction of new dosage forms (Rezvanian et al., 2017).

At present, the hydrogels used in film formulations include chitosan, sodium alginate, polyvinyl alcohol, polyurethane (PU), etc (Tavakoli and Klar., 2020). The polyelectrolyte composite membrane made of *Bletilla striata* polysaccharide combined with chitosan by Wang et al. has sustained-release properties, which can increase the drug concentration at the lesion, which is helpful for the treatment of oral ulcers (Wang et al., 2020). Guo et al. used Ca^2+^ as a cross-linking agent and glycerol as a moisturizing agent to make the asiaticoside sodium alginate repair patch, which has the effect of inhibiting the high expression of pro-inflammatory factors and promoting the formation of collagen fibers in the wound tissue. At the same time, it can reduce the local inflammation of the wound, thereby promoting the repair of the wound and shortening the healing time of the wound skin (Guo et al., 2020).

In general, the gel film has good biocompatibility and tissue adhesion, is easy to use due to its elasticity and flexibility, allows proper oxygen and water exchange during wound healing. And it is able to absorb the serous secretions of lesions, reducing the interference with the wound healing process, which is of great help in wound healing.

The four types listed above are common types of TCM films. In addition, there are acupoint application. The difference between TCM film preparations and chemical transdermal preparations is that the former not only exerts curative effects locally on the skin or enters the body through the skin, but also exerts curative effects through meridian points (Li et al., 2022). For some special TCM film, it is under the guidance of TCM theory, the medicine is pasted on certain acupoints of the human body. Through the percutaneous absorption of the drug, it not only stimulates the local meridian points, but also stimulates the whole-body meridian to prevent and treat diseases (Xue et al., 2022). Zhao et al. used the oral traditional Chinese medicine decoction prepared by red peony, Chuanxiong rhizome, etc., combined with acupoint sticking prepared by *Evodia*, *cassia* twig, etc. to treat tubal infertility. They found that taking traditional Chinese medicine decoction can achieve the effects of removing blood stasis and clearing the meridians, promoting blood circulation and relieving pain, warming the meridians and dispelling cold. The acupoint application uses the point of view of acupuncture and moxibustion of traditional Chinese medicine, and selects the four acupoints of Zhongji*,* Zhongwan, Qihai and Tianshu for drug application, which can promote the absorption and introduction of drugs, achieve the effect of reconciling the meridians and collaterals, nourishing blood and consolidating the root. It can be seen that the traditional Chinese medicine decoction combined with acupoint sticking combined with tubal drainage has better efficacy in the treatment of tubal infertility (Zhao et al., 2021). The film prepared by Liu et al. using TCMs such as *Scutellaria baicalensis* and *Ephedra* has the effect of relieving cough and relieving asthma. Through acupoint percutaneous administration, it was found that the skin resistivity rate decreased, the Na^+^-K^+^-ATPase activity increased, and the transdermal absorption increased after administration, which was significantly different from that of non-acupoint administration (Liu et al., 2000). In a word, the traditional Chinese medicine film administered through acupoints can make the medicine reach the lesions directly, and can also enhance the regulating effect of the body through the stimulation of acupoints, and obviously enhance the therapeutic effect. At the same time, it has the advantages of less toxic and side effects and long drug release time, which can reduce the shortage of oral administration.

## 3 The composition of raw materials of TCM film

TCM films are generally composed of active ingredients of TCM and film-forming materials, and some films also contain film-forming auxiliary materials such as penetration enhancers and pressure-sensitive agents.

### 3.1 Active ingredients of traditional Chinese medicine in film

TCM refers to the drugs collected, processed and prepared under the guidance of TCM theory to explain the mechanism of action and guide clinical application. It is the main component of TCM films. TCM film refers to the film dosage form made of the active ingredients or their extracts obtained from a single or compound TCM through a reasonable process and suitable excipients or substrates under the guidance of the theory of TCM. TCM compound is an organic combination of multiple TCMs for specific diseases, which is a major feature of TCM treatment. For the TCM compound itself: the TCM compound has many flavors, complex components, most of the pharmacodynamic chemicals basis is not clear, and the amount of most active ingredients in the medicinal components is relatively low, which also brings great difficulties to the determination of active ingredients. In addition, the active ingredients of medicinal materials may vary greatly due to factors such as origin, climate, processing technology, etc., so this brings greater challenges to the quality control of compound recipes composed of different medicinal flavors (Li et al., 2005). In the research of TCM film preparation, reasonable methods and processes should be selected as far as possible according to the composition and compatibility of the original prescription. It is necessary to pay attention not only to in-depth chemical, pharmacy and clinical research on the original formula, including pharmacokinetic research, but also to combine the requirements for approval of new drugs, and try to avoid the use of drug bases containing heavy metals (Yuan et al., 2003). In TCM treatment, after the film is administered through the skin and cavity, it not only avoids the first-pass effect of the liver, but also reduces the irritation and adverse reactions of the drug to the gastrointestinal tract, and improves the bioavailability (Wang et al., 2017).

TCM mainly comes from natural medicine and its processed products, including botanical medicine, animal medicine, mineral medicine and some chemical and biological products. The ingredients of TCM are complex and diverse, mild and non-irritating, and have few side effects. In recent years, the research on TCM films has found that TCM can not only function as their active ingredients, but also serve as film-forming materials, penetration enhancers, colorants, and flavoring agents (sweeteners, aromatics, Acidic agents, effervescent agents and bitterness inhibitors, etc.) are involved in the preparation of film formulations to achieve “medicine and supplementary integration”. For example, in the article of Zhu et al., it is correctly pointed out that the composition of the dosage form that *Bletilla Striata* Gum participates in, as the main drug, it can be used for the preparation of film coating agents, etc., as a pharmaceutical excipient, it can be used as a gel matrix, and the film-forming material is further prepared Gels and films (Zhu et al., 2019). Chen et al. stated that the volatile oil of TCM has good transdermal absorption characteristics, such as *Asarum*, cinnamon, etc., and can be used as a penetration enhancer to participate in the preparation of film preparations (Chen et al., 2016). As a natural pigment, TCM not only has a wide range of sources, but also has low harm. While it could be safe and reliable as medicine uses for multiple purposes with special dosage and preparation (Zhang et al., 2007). The konjac glucomannan-based gardenia yellow pigment film prepared by (Liu and Cui. 2019) has good performance and stable colour. This study shows that TCM can be used as a colorant to participate in the preparation of the film (Liu et al., 2021). In addition, TCM can also be used as a flavoring agent to participate in the preparation of various dosage forms, such as honey, mint, fennel, musk, etc (Xiao et al., 2021). The above content shows that Chinese herbal medicines are not limited to exerting a single effect, but have multiple effects.

The therapeutic effect of the film is empowered by the composition of TCM, most of which are plants and cannot be directly used as medicine. It needs to be used as medicine by decocting or concocting to extract the active ingredients in TCM. The active ingredient of TCM is the key factors for the film to exert its curative effect. It is mainly composed of active ingredients of TCM decoction or powder medicine that are not suitable for decoction. We selected some representative TCM films and listed their active ingredients, indications and pharmacological effects of the main TCM (Table 2).

**TABLE 2:** Traditional Chinese medicine components and pharmacological action of film agent.

Name of film agent	Active ingredients of films	Indications	Pharmacological effects of main Chinese medicines
Bletilla Oral Ulcer Film (Zhang, Sun et al., 2014)	*Bletilla*	Oral Ulcer	Traumatic bleeding due to the efficacy of arresting bleeding with astringent action (He et al., 2017)
Indigo Naturalis Film (Fan et al., 2008)	Indigo naturalis, Borneol	Oral Ulcer	Indigo Naturalis has the effect of clearing heat and detoxifying, cooling blood and eliminating spots, reducing fire, and calming shock (Fan et al., 2008)
Oral Ulcer Double-layer Membranes (Cheng et al., 2017)	Cortex Phellodendri	Oral Ulcer	Cortex Phellodendri has antibacterial and anti-inflammatory effects, as well as obvious antifungal and immunomodulatory effects (Kitt et al., 2015).
Lavender Essential Oil Film (Wu, 2021)	Lavender	Burns	Lavender essential oil has the functions of drying dampness, relieving pain, purging fire and detoxifyingm (Wu, 2021)
“Ophthalmic Washing Solution No.1” Chinese traditional medicine liniment (Zhang et al., 2016)	*Rheum officinale, Coptis Chinensis, Scutellaria baicalensis, Phellodendron*	External eye disorder caused by heat toxin	Rheum Officinale, Coptis Chinensis, Scutellaria baicalensis, Phellodendron have heat-clearing and detoxifying effects (Zhang et al., 2016)
Qumin Tongbi Nasal Spraying Agent (Chen et al., 2011)	*Astragalus membranaceus*, Flower bud of lily magnolia, Smoked plum, *Lithospermum*, *Asarum*, *Eclipta alba*	Perennial allergic rhinitis	Several drugs cooperate with each other, tonifying lung and kidney, dispelling cold (Chen et al., 2011)
Erhuangsan Bletillae Rhizoma Gelatin Sustained Release Double-layer Membrane (Wang et al., 2019)	*Bletilla*, *Acacia catechu*, *Coptis chinensis*, Alum	Treatment of cervical precancerous lesions	Bletilla has the effects of antibacterial and anti-inflammatory, removing saprophytic muscle, reducing drug toxicity and anti-tumorm (Chen et al., 2011)
Whitening and Moisturizing Facial Mask (Zhou et al., 2019)	*Angelica*, *Poria*	Whitening and moisturizing	Angelica and Poria have the ability to eliminate excessive pigment accumulation in tissues Ability to promote skin cell metabolism (Zhou et al., 2019)
New Compound Chinese Medicine Coating Agent (Wang et al., 2018)	*Creeping oxalis*, *Poria, Achyranthes bidentata*, *Forsythia suspensa*, *Menthol*, *etc*	Skin scar	Creeping oxalis has an antioxidant effect and can inhibit the inflammation of scars. *Achyranthes* bidentata and Forsythia suspensa have anti-inflammatory and detumescent effects (Zhou et al., 2019)

### 3.2 Film-forming materials

The film-forming material is the carrier of TCM ingredients, and its physical and chemical properties are stable and do not react with TCM ingredients. It has the characteristics of non-toxicity, no side effects, good film-forming and film-releasing, and low cost. It can be divided into natural polymer film-forming materials and synthetic polymer film-forming materials.

In general, most of the common traditional Chinese medicine films are mainly used in the above two categories of film-forming materials. With the deepening of research on nanomaterials, many nanomaterials have been used in drug delivery systems, such as liposomes, transfersomes, alcohol liposomes, nanoparticles and copolymer carriers (Wang et al., 2016). Due to the small particle size of nano-drugs and their good effects in drug retention and specific targeting, new types of nano-material-based membrane agents are gradually being used in the treatment of clinical diseases. Nunes et al. provided an efficient method for the development of wound dressing materials with enhanced properties based on biocompatible chitosan and poly (vinyl alcohol) hydrogels with embedded silver nanoparticles as a potent antimicrobial agent (Nunes et al., 2011). However, few nanomaterials have been used in traditional Chinese medicine films in the current. It is believed that with continuous research, more nanomaterials will be used in traditional Chinese medicine film drug delivery systems, so that the film drug system can play a more powerful effect.

#### 3.2.1 Natural polymer film-forming material

The commonly used natural polymer film-forming materials are starch, dextrin, cellulose, chitosan, agar, gum arabic, sodium alginate, etc. Cellulose is the main component of plant cell walls, which is non-toxic, biodegradable, hydrophilic, and biocompatible. The cellulose extracted from plants shows excellent properties, making it suitable for the pharmaceutical industry (Naomi et al., 2020).

Chitosan and chitin have similar chemical structures with cellulose. Chitosan is the product of N-deacetylation of chitin and completely non-toxic, so it could be safely used in oral. Besides, it has several advantages in biomedical applications, such as biocompatibility and controlled biodegradability, which can produce degradation products that are non-toxic and do not generate inflammatory responses. In addition, chitosan can control drug delivery for use as wound dressings due to its unique polycationic, nontoxic, antibacterial, and bioabsorbable properties (Muxika et al., 2017). It also has good film-forming properties after being dissolved, and has good biocompatibility with the human body. Therefore, the chitosan is currently recognized as a natural polymer film-forming material with great potential for development.

Sodium alginate is a by-product of extracting iodine and mannitol from the brown algae kelp or *Sargasso*. It contains a large amount of acetate, which can show polyanionic behavior in aqueous solution and has certain adhesion. This chemical is popular be used as a drug carrier for the treatment of mucosal tissues (Moebus et al., 2012). It can enhance the viscosity of liquid medicine without toxicity, and the film-forming conditions are relatively stable, so it is a good film-forming material.

#### 3.2.2 Synthetic polymer film-forming materials

At present, synthetic polymer film-forming materials can be classified with three categories according to their molecular structure: polyvinyl alcohol compounds, acrylic copolymers and cellulose derivatives.

##### 3.2.2.1 Polyvinyl alcohol compounds

Polyvinyl alcohol (PVA) is one of the widely studied synthetic polymers for biomedical applications, especially the fabrication of wound dressings based on nanofiber membranes, which are biocompatible, biodegradable, electrostatically spin-dry, hydrophilic properties, bioadhesives, non-toxicity and chemical resistance (Kamoun, et al., 2021). Therefore, PVA is a good film-forming material and is widely used in film formulations. At present, medical polyvinyl alcohol has specifications such as PVA05-88, PVA17-8 and PVA-124 (Zhang et al., 2004).

##### 3.2.2.2 Acrylic copolymers

Among acrylic copolymers, polyacrylate and carbomer have good film-forming properties. Polyacrylate can form a film agent with good gloss and strong water resistance, which has good adhesion, good flexibility, elasticity, and good weather resistance, but poor pull resistance. Carbopol is a polymer of acrylic acid-bonded allyl sucrose or pentaerythritol allyl ether. It is a white loose powder, soluble in water and organic matters of glycerol and ethanol, and has excellent film-forming properties and adhesion. Liu et al. made Shuanghuang gel with carbomer-940, which has good transdermal absorption ability, no greasy feeling, even smear, easy cleaning, good coupling with the skin, and high bioavailability (Liu et al., 2019).

##### 3.2.2.2 Cellulose derivatives

Cellulose derivatives of carboxymethyl cellulose (CMC), hydroxypropyl methylcellulose (HPMC) and methylcellulose (MC) are common food additives. They are attached to the main chain of anhydrous glucose to make their corresponding cellulose derivatives show hydrophilicity and water solubility for enhancing properties of film-forming and releasing (He et al., 2021). Hydroxypropyl methylcellulose (HPMC) and sodium carboxymethyl cellulose (CMC-NA) are film-forming materials of cellulose derivatives commonly used in TCM. As a water-soluble polymer material, HPMC has good water solubility, dispersion, thickening, water retention and film-forming properties. The formed coating film is colorless, odorless, tough and transparent, and is widely used as drug coating and rate-controlling polymer materials for sustained release formulations (Wang, 2018). Sodium Carboxymethyl Cellulose (CMC-Na) is normally a kind of powder, granular or fibrous substance with white to light yellow colour. Due to its strong hygroscopicity, soluble in water, and certain thickening and adhesive properties, it is easy to form a film. CMC-Na was used as a film-forming material in the preparation of oral patches and the obtained patch is smooth without bubbles, flexible and uniform, and has excellent adhesion properties (Chen et al., 2020).

### 3.3 Other auxiliary materials

In order to improve the performance of the film and facilitate storage, some film formulations also add auxiliary materials such as penetration aids, pressure sensitive agents, preservatives, plasticizers, etc.

#### 3.3.1 Penetration enhancer

Penetration enhancers are substances that increase or accelerate the penetration of a drug through the skin. The ideal penetration enhancer has no irritation or damage to the skin and mucous membranes, and is physically and chemically stable. In addition, it can also promote faster transdermal delivery of Chinese herbal ingredients without reacting with them. Penetration enhancers can be classified into alcohols and polyols, lactams and their analogs, esters and ethers, surfactants, fatty acids, terpenes, steroids, and miscellaneous items according to the molecular structure (Vasyuchenko et al., 2021). The commonly used penetration enhancers in TCM films are azone, linoleic acid, propylene glycol, borneol, menthol and wormwood, etc. Chen et al. focused on the effects of propylene glycol, azone, menthol and oleic acid on the transdermal absorption of evodiol base in the preparation of the Evodiamine Hydrogel Patches, among which propylene glycol has the better effect of promoting penetration (Chen et al., 2021).

#### 3.3.2 Pressure-sensitive agent

Pressure sensitive agent is an important auxiliary material of TCM film, which belongs to viscose material. It enables the film to have strong adhesion under a slight pressing force but also easy to peel off. According to its composition, pressure-sensitive adhesives can be divided into the following categories: polyisobutylene pressure-sensitive adhesives, polyacrylate pressure-sensitive adhesives, cinnamon rubber pressure-sensitive adhesives, cinnamone pressure-sensitive adhesives, hot-melt pressure-sensitive adhesives, etc (Ke et al., 2018).

#### 3.3.3 Plasticizer

The plasticizer is a kind of material that can reduce the rigidity and brittleness of polymer. Plasticizers in the film-forming system can increase the flexibility and ductility of dry film and prevent the rupture of dry film, and some plasticizers can also promote permeability (Asghar, 2020). Plasticizers commonly used in TCM films include glycerol, dibutyl sebacate, sorbitol, and so on. Wang et al. added an appropriate amount of glycerol in the preparation of Spray Film with Clematis Root, and the obtained film spray had good film-forming properties, and had good hygroscopicity, moisture retention and air permeability (Wang et al., 2019).

#### 3.3.4 Preservatives

The preservative is a kind of food additive that can inhibit the activity of microorganisms and prevent the spoilage of food. Preservatives added to the film agent can effectively prevent the film agent from spoilage. A large number of studies have pointed out that the main preservative of the coating agent is paraben ester series, which could increase the stability of the liquid preparation and extend the shelf life (Jin et al., 2012). Paraben is a white crystalline powder or colorless crystal, easily soluble in alcohol, ether and acetone, very slightly soluble in water, and can be used as a bactericidal preservative in TCM films. Sorbic acid, benzyl alcohol, paraben, etc. are commonly used preservatives in TCM. Cai et al. added an appropriate amount of ethyl paraben as a preservative in the preparation of the compound sore film, so that the film can be stored for a long time (Cai et al., 2012).

In short, the above list is the composition of common traditional Chinese medicine film preparations. Interestingly, some Chinese medicinal active ingredients have their special film-forming properties. For example, konjac glucomannan (KGM), white jelly polysaccharide, etc. have superior self-film-forming properties. Using it in the film has the effect of killing two birds with one stone (Dong et al., 2019; Liu et al., 2021). In addition, polymers such as S-nitrosoglutathione (GSNO), dialdehyde starch, titanium dioxide and whey protein isolate, zein, iodine, etc. can be seen in some chemical films different from traditional Chinese medicine films. Unique membrane components like copper (CuI) nanoparticle dispersions, liposomes, etc. It has been reported that by using chitosan and GSNO to make a film-type dressing that can release NO, it has better sterilization and wound healing effects (Kim et al., 2015). Alizadeh San et al. used dialdehyde starch as a cross-linking agent to optimize the properties of films made from a mixture of collagen, hyaluronic acid, and chitosan, making them smoother, more elastic, and resistant to breakage (Sionkowska et al., 2020). Furthermore, nanocomposite films are widely used due to their controlled release of antimicrobial compounds and their biodegradability. For example, whey protein isolate, cellulose nanofibers, TiO2 nanoparticles, and rosemary essential oil nanocomposite films have significant broad-spectrum bioinhibitory effects and can be used as safe and good biological preservatives (Alizadeh Sani et al., 2017). Studies have shown that the addition of zein to a single-layer film coating for colon-targeted drug delivery can protect the active agent from degradation and early release in the stomach and small intestine, improving the drug-targeted transport capacity in colonic delivery systems (Nguyen et al., 2019). Applying CuI nanoparticle dispersions to films and fabrics can be used as anti-SARS-CoV-2 materials by virtue of their ability to release copper ions with high virucidal ability (Takeda et al., 2021). Studies have shown that liposomes, as a biocompatible and biodegradable drug delivery system, can encapsulate lipophilic drugs and release drugs slowly and continuously at the lesion site. And it has less cytotoxic effect on normal tissue, and is a good carrier of tumor chemotherapy drugs (Wang et al., 2019). The above film components show their unique advantages and effects in chemical medicine films. It is expected that the advantages of such materials can be fully absorbed in the future development of traditional Chinese medicine films, so as to make the best use of them and complement each other.

## 4 Preparation process of TCM film

**FIGURE 3:**
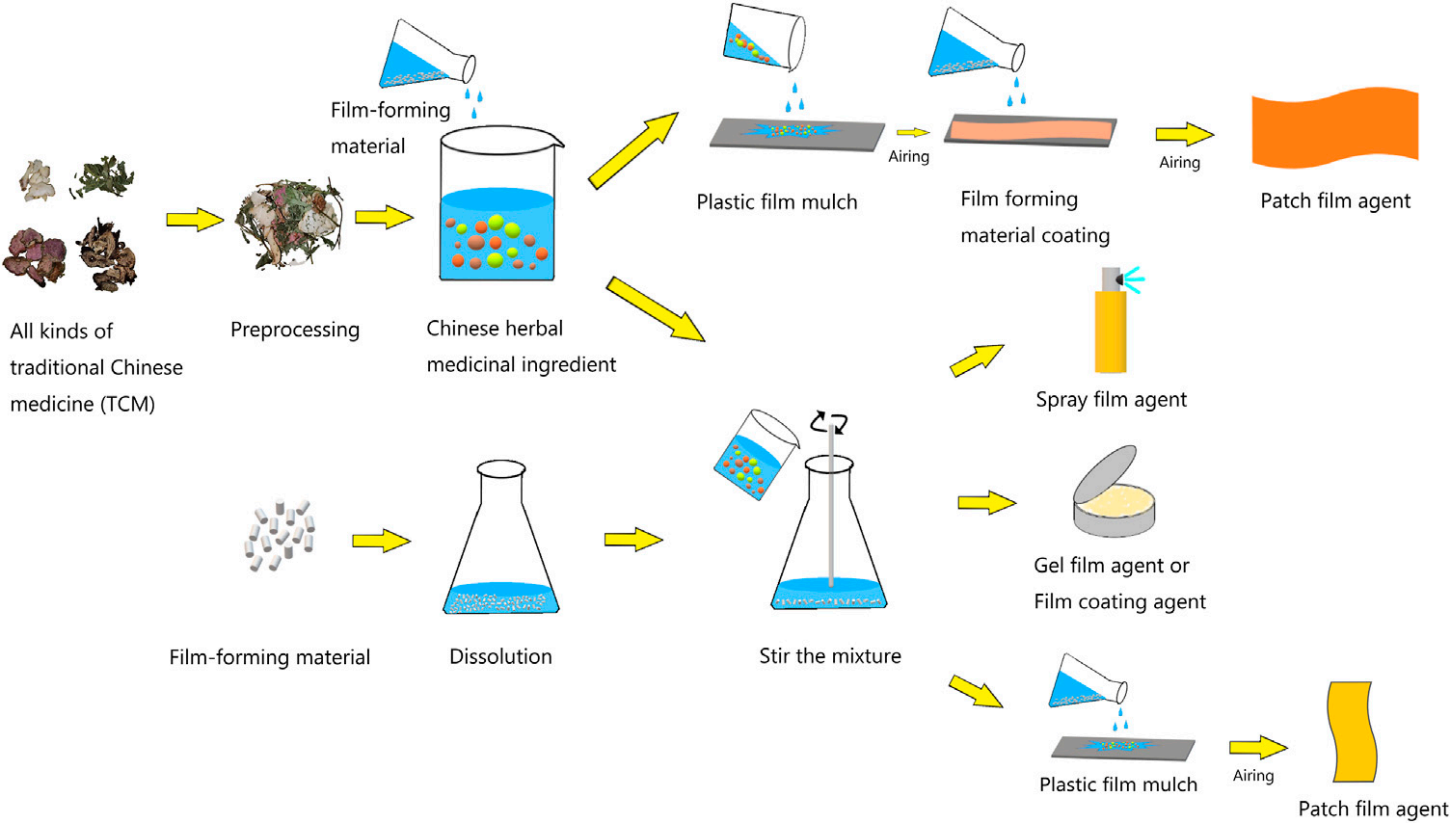
Preparation process of TCM film agent.

There are many kinds of TCM film agents, but the preparation process is roughly similar, which can be divided into the preparation of TCM components and film agents. The film preparation can be divided into two parts: the dissolution of the film-forming material and the mixing of the TCM and the film-forming material (Figure 3).

### 4.1 Preparation of TCM ingredients

**FIGURE 4:**
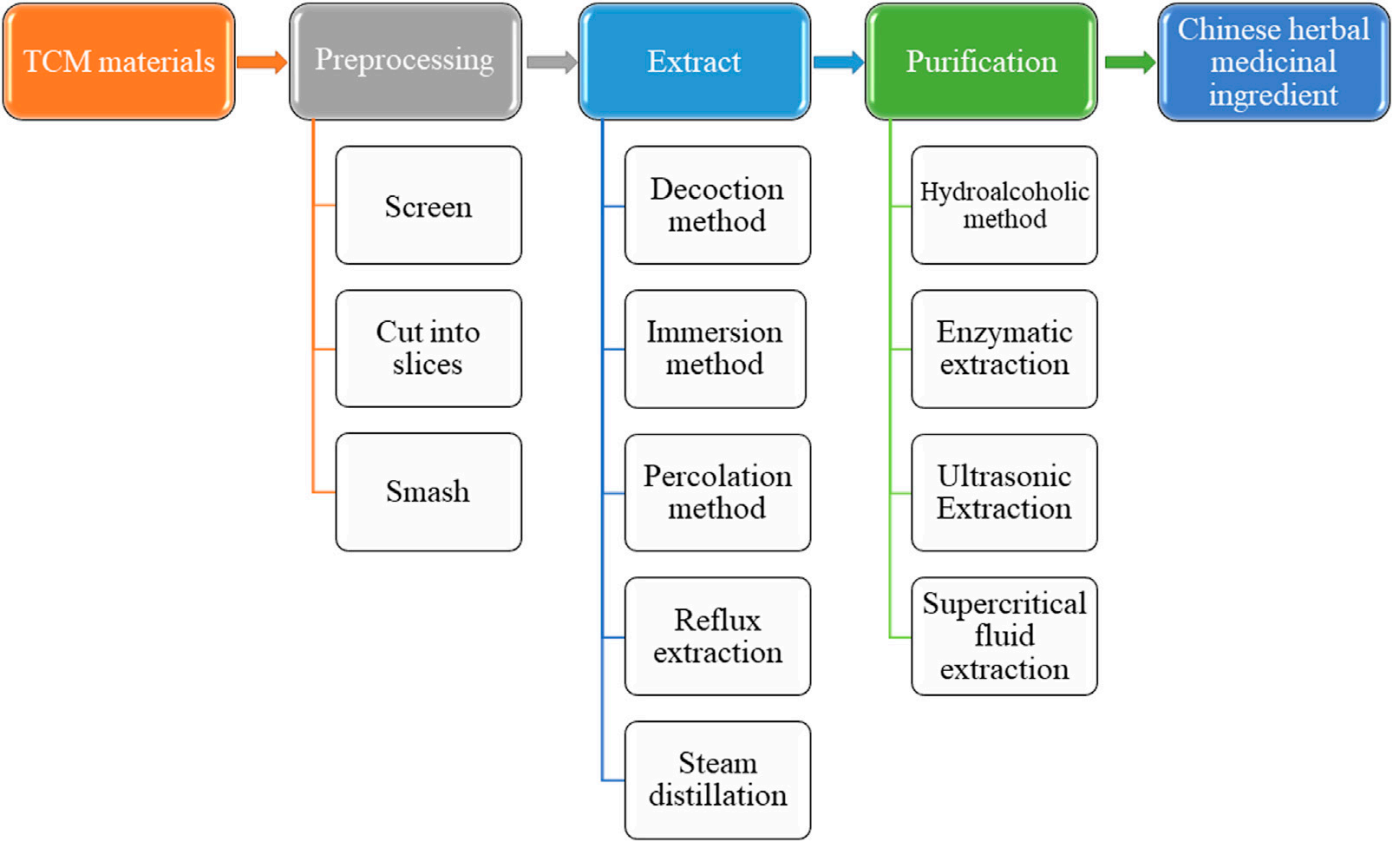
Preparation of TCM ingredients.

The traditional methods for extracting ingredients from TCM include decoction, immersion, percolation, reflux extraction, steam distillation, etc. The hydroalcoholic method is commonly used for purification. With the continuous introduction of new technologies, several new approaches like enzymatic extraction, ultrasonic extraction, supercritical fluid extraction and soon are applied (Figure 4), to reduce the impurities (Hou and Li., 2011).

### 4.2 Preparation of film agent

When preparing a film agent, usually the film-forming material should be fully dissolved with water or ethanol first, and then the preparation method will be different depending on the type of film agent. After the film preparation is completed, strict performance tests are also carried out for screening and perfecting the formula.

Film coating agent, spray film agent and gel film agent are prepared by mixing film-forming materials with TCM ingredients. For example, when preparing Chushi Tongluo film coating agent, take appropriate amounts of CMC-Na and PVA-124, respectively add distilled water to swell, heat to swell into a gel, mix and stir evenly, and serve as a matrix for use. Then take the medicinal extract of the formula amount and add it to the base with slow stirring (Wu et al., 2019). The patch film is first mixed with the TCM ingredients and some auxiliary film-forming materials, and then spread on a glass plate or petri dish to dry. Then the film-forming materials are spread on the dried TCM ingredients, and finally the preparation is completed by drying.

Among them, Scarpa et al. summarized the thin film casting of solutions, suspensions or melts, usually by solvent casting, semi-solid casting, rolling, coating, and hot-melt sheet extrusion (Scarpa et al., 2017). At the same time, different preparation methods are used when preparing some chemical membranes. Wang et al. use electrostatic spinning legal preparation of dexamethasone palmitate-loaded electrospun nanofiber membrane for ocular application. It has the advantages of equipment and experimental costs, high fiber production rate, and effectively increase the absorption of drugs (Wang et al., 2020). In addition, Varan et al. cervical films prepared using inkjet printing technology show that printing technology may be suitable for the development of biological viscosity membrane combinations of antiviral and anticancer drug loads. The amount of drugs can also be controlled and modified in a specific way of patients. In addition, the diaphragm made by inkjet printing can obtain extended drug release time (Varan et al., 2017).

The formulation detailed of oral patch film by Jacob et al. is a worthy reference of the film preparation. Its commonly used film casting method is the solvent casting method, which begins with precise dispensing of the drug, adding the solvents in the proper sequence to a thermostatically controlled mixer. Then mix with an appropriate high or low shear mixer to ensure homogeneity (Jacob et al., 2021). However, the dose usually prepared in the laboratory is small, and there is no large-scale machine, but the coating method, casting method, and glue injection method are commonly used.

Pre-clinical film product development includes rigorous physical and chemical testing to assess product functionality and consistency. Standard tests for film formulations include drug content, drug content uniformity, contact angle, water content, solubility, disintegration, as well as tensile strength, puncture strength, elongation, young’s modulus, folding endurance, etc (Bala et al., 2013). Among them, dosage form disintegration and dissolution are two of the most critical parameters for determining the effective level of a drug at the desired site. Grab et al. developed a quantitative texture analyser method that reduces user bias and standardizes testing, which is accurate and predictable. The disintegration time of the vaginal membrane is measured repeatedly and can also be modified to apply to other membrane types (Grab and Rohan., 2018).

The film formulation will finally complete the delivery of the active ingredients of the drug to the clinic through preparation design, absorption and release, and clinical application composed of raw materials and matrix (Ma et al., 2022). During the period, the evaluation of the efficacy of each link can reflect the overall quality of the film. It can be roughly divided into *in vivo* and *in vitro* consistency evaluation. *In vitro* includes formulation properties, *in vitro* release properties and *in vitro* transdermal properties (Zhu et al., 2020). Among them, the *in vitro* release test (IVRT) can characterize the drug solubility, particle size, etc., distinguish the difference in release ability between film agents, or evaluate the influence of small differences in the preparation process on the performance of the preparation. The most classic and convenient method is the diffusion cell method (Shao et al., 2021). In addition, emerging research methods of pharmaceutical rheology can reflect the dynamic properties of various films including flow behavior, viscoelasticity and deformation behavior. This reflects the external characteristics of the film itself, such as the viscosity of the film itself, the degree of adhesion to the medicinal site and the storage stability, and the volume and shape changes in response to external forces (Liu et al., 2022). The *in vivo* consistency evaluation pays more attention to the clinical safety and efficacy of film preparations, including studies on the equivalence of clinical endpoints, the equivalence of pharmacodynamic endpoints, the equivalence of pharmacokinetic endpoints, and skin pharmacokinetics (Zhu et al., 2020).

## 5 Clinical application

With the continuous development of research, the clinical contribution of TCM is becoming more and more prominent. The philosophy of TCM presents a holistic and systematic view of the treatment of various diseases. Its characteristic is to reach multiple targets and affect various pathways through synergistic therapeutic effects. Normalizes functions and maintains the balance of “yin” and “yang” in the body. In addition, TCM also has a two-way regulating effect. Common forms include regulating “yin” and “yang,” cold and heat, internal and external, inhibition and stimulation, blood circulation and blood disorders. Thus, exerting a more powerful therapeutic effect than a single ingredient (Peng et al., 2022). The application of these films is gradually discovered and confirmed for treating various ulcers, skin diseases, skin repair, local inflammation and gynecological disease. In recent years, more scholars have found that TCM film agent can be further applied to other clinical diseases, which reflects that film agent has great clinical application value. At present, the research on TCM film is focusing on elucidating the effective mechanism of its efficacy, in order to provide a theoretical basis for the application of this medicine.

### 5.1 Treatment of ulcers

#### 5.1.1 Oral ulcer

Oral ulcer is a common mucosal disease in the clinic and its pathogenesis is still unclear. It has the characteristics of limitation, self-healing effect, recurrence, severe wound pain. Thus, oral ulcer has a significant impact on the quality of life of patients. At present, more and more TCM films are used in the treatment of oral ulcer, greatly relieve the pain of patients. Because it can not only form a moist and airtight space on the ulcer surface to isolate the complex environment in the oral cavity, but also make the medicinal ingredients infiltrate the wound stably and continuously to improve the curative effect (Table 3). At present, a large number of studies have shown that traditional Chinese medicine may treat oral ulcers by affecting the NF-κB inflammatory pathway and the PTEN/AKT/GSK3β pathway (Gao et al., 2021; Zheng et al., 2021).

**TABLE 3:** Commonly used oral ulcer patch formula and pharmacological mechanism.

Name of film agent	Major Chinese medicines	Pharmic function
Okra Flavonoids Oral Ulcer Film(Zhang and Chen, 2018)	Okra Flavonoids (Li et al., 2021)	Okra flavonoids have antipyretic, detoxifying, anti-inflammatory, and bacteriostatic and analgesic properties
Yang Yin Sheng Ji Pulvis (Hu et al., 2019)	Indigo naturalis, Cortex Phellodendri, Catechu, Mentha, Gentian, Bezoar, Glycyrrhiza	Bezoar detoxification, cool and refreshing, with oral pharynx sore swelling poison, pain, and decay effect
		Gentian purifies fire and dampness of the liver
		Licorice has a healing ulcer, detoxification anti-inflammatory effect
Xiaobo Muti Oral Ulcer Film (Wang et al., 2017)	*Berberis amurensis*	Barberry has the effect of clearing heat and dampness, anti-inflammatory and analgesic, reduce
		The crude extract from the fruiting body of the fungus of lamella planta had anti-inflammatory and analgesic effects
Oral Ulcer Double-layer Membrane (Cheng et al., 2017)	Cortex Phellodendri	Phellodendron has the effect of clearing heat and detoxifying, detoxifying, and treating sores
*Periplaneta americana* Oral Film (Li et al., 2018)	Abstract of *Periplaneta americana* L.	Periplaneta Americana can enhance immunity, promote wound healing, tissue repair and improve microcirculation
Double-layer Drug Films Of Compound Resin Draconis (Zhang et al., 2017)	Dragon’s blood, *Coptis chinensis*	Dragon blood exhaust, coptis have the effect of activating blood and relieving pain

#### 5.1.2 Chronic skin ulcer

Chronic skin ulcer (CSU) are skin defects caused by various causes and commonly seen in patients with leprosy and diabetes. Besides, CSU has a tendency of cancerization if it is not cured on time and it has a possibility of recurrence after curing. At present, some studies have confirmed that some TCM compound films have a certain curative effect on CSU. For example, the Shixiang plaster can promote ulcer healing by inducing angiogenesis in wound granulation tissue in diabetic foot ulcers (Fei et al., 2019). Moreover, chitosan, tea polyphenol and vitamin B_12_ were used to prepare the compound chitosan film. And researchers proved that the therapeutic effect of the film group was much higher than the control group by establishing an animal model with rabbit, providing a strong basis for clinical application of the film (Guo et al., 2007). TCM may repair the mucosa by regulating the NLRP3/Caspase-1 signaling pathway and the TGF-β1/p38MAPK signaling pathway, thereby treating skin ulcers (Huang and Wang., 2022; Xu et al., 2022).

### 5.2 Treatment of skin disease

As the first defense against bacteria and viruses of body, the skin is directly exposing to the outside world. Therefore, when subjected to various strong stimuli, the skin barrier is easy to be broken-down. On the other hand, the scars left by improper treatment of damaged skin tissue can seriously affect the appearance of the skin. These have both physical and psychological effects on the patient.

In recent years, more and more TCM patches have been widely used in clinical treatment of skin diseases such as warts and acne, and the effect is very remarkable. For example, through analysis on treatment of facial condyloma latum in 110 cases by Chinese herbal mask, Guo and colleagues found that feature an efficacy method. And the results showed that the effective rate was 92.63%, which has clinical promotion value (Guo and Lu., 2006). And in 2018, a statistical comparison conducted for exploring the therapeutic effect of TCM mask on acne found that TCM film has a good anti-inflammatory effect and can effectively cure acne (Ma et al., 2018). Furthermore, the application of TCM film in the treatment of skin burns and scar repair is gradually accepted. For example, Bo et al., made a burn type II spray film with a variety of TCMs such as *Radix Scutellaria*, safflower and *Cortex phellodendron*, and then a disinfectant spray film appeared in 2020, both of which can prove that the TCM film can indeed be used to treat burns and scalds (Dang et al., 2019; Ju et al., 2020). And referring to the comparative study of Wang et al., it was observed that the new compound TCM coating agent can effectively treat skin scars, and it was found that TCM can effectively inhibit the proliferation of fibroblasts (Wang et al., 2018). All indicated that TCM has a good therapeutic effect in the treatment of skin tissue damage. Studies have shown that the treatment of such skin diseases by TCM is achieved by regulating the TLR-2/NF-κB pathway, the TGF-β1/Smads signaling pathway, and the targeting of miR-21 to regulate the mTOR pathway (Ai et al., 2021; Tang et al., 2021; Xue et al., 2021).

### 5.3 Body care

TCM mask is a common coating formulation of TCM cosmetics. For people pursuing green, safe and high-effective beauty products, it is more inclined to develop a compound TCM mask based on doctor’s prescription and penetrate directly to the skin. To improve wrinkles, chloasma, dry skin, dark yellow after long acne, facial pigmentation after burns and other common skin problems (Lei et al., 2010). For instance, the chitosan TCM mask made of *Angelica dahurica*, *Scutellaria baicalensis*, *Cape jasmine*, *Honeysuckle* and dandelion in a certain proportion. Then let 30 patients with dry skin, dark color and severe pigmentation use it for 1 month to observe the effect. The results showed that the mask has good antibacterial, whitening and moisturizing functions. And it is a new type of TCM mask with natural materials and no side effects (Xu et al., 2011). Studies have shown that the whitening effect of TCM may be managed through signaling pathways of cyclic adenosine monophosphate (cAMP)/protein kinase A (PKA)/response element binding protein (CREB), mitogen-activated protein kinase (MAPK), stem cell Factor (SCF)/c-Kit receptor, secreted glycoprotein (Wnt) etc. (Yang et al., 2021).

### 5.4 Local inflammation

Inflammation refers to the defensive response of living tissue with vascular system to the stimulation of various damage factors. But under certain circumstances, the body’s excessive inflammatory response can cause serious damage to itself. Common local inflammatory diseases include arthritis, endophthalmitis, periodontitis, and psoriasis. The anti-inflammatory effects of herbal extracts and their related films have been extensively studied. A variety of inflammation models have confirmed that both the external treatment of TCM films and the combination of Chinese and Western medicine have a positive effect on this kind of inflammation (Li et al., 2007; Liu et al., 2019; Qiu et al., 2021).

In Wang’s report, 60 patients were applied for evaluating the clinical effect of Xueshan Jinluohan analgesic film combined with diclofenac sodium sustained-release tablets in the treatment of acute gouty arthritis. It was found that the total clinical effective rate of the treatment group using the combination therapy was 86.67%, while that of the control group was only 70.00%. Thus, it refers that the use of this film to intervene in acute gouty arthritis can reduce inflammatory damage, relieve joint pain and swelling, and promote the recovery of joint function (Wang and Liu, 2018).

In the report on the treatment of endophthalmitis, the experiments by constructing a rabbit model of bacterial endophthalmitis showed that implantation of chitosan film into the suprachoroidal space could allow the drug to be absorbed from the abundant blood vessels in the choroid. Not only reduce the toxic and side effects of drugs on the retina, but also avoid the blocking effect of iris on the absorption of medicinal components. Provide experimental basis for clinical application (Li et al., 2007). Studies have confirmed that traditional Chinese medicine may play an anti-inflammatory role by acting on the COX-2/NF-κB pathway and Wnt/β-catenin and other signaling pathways (Zhang et al., 2021; Xiao et al., 2022).

### 5.5 Gynecological diseases

By application of TCM preparations in gynecological diseases such as cervical erosion, cervical cancer, primary dysmenorrhea, it found that the administration of films through the vaginal mucosa can increase the area of drug action, improve the utilization rate of drugs, which opened a new way on therapy gynecological diseases (Yu et al., 2018). For instance, in order to prolong the drug retention time and reduce the number of dressing changes, Wang et al., improved Erhuang Powder into a film, which has a satisfactory effect on the treatment of cervical intraepithelial neoplasia (CIN). And the film provides a feasible modern Chinese medicine preparation for treating cervical cancer (Wang et al., 2019). Studies have shown that TCM can intervene in diseases by regulating the NF-κBTGF-β1Smad pathway and endometrial receptivity-related signaling pathways such as Wnt, MAPK and STAT3 (Hu et al., 2020; Zhao et al., 2021).

### 5.6 Other clinical diseases

In addition to the clinical application of TCM film agents in the above studies, it is also involved in other aspects, but there are few reports on relevant studies at present (Table 4).

**TABLE 4:** Other clinical application and pharmacological mechanism of Chinese medicine film agent.

Name of film agent	Major Chinese medicines	Application	Pharmic function
Dragon’s Blood Gel (Shi et al., 2019)	Dragon’s blood, *Bletilla*	Bedsore infection	Dragon’s blood is used externally to nourish muscle and collect sores, remove stasis and detumescence
Chushi Tongluo Linimen (Wu et al., 2019)	radix *Aconiti Kusnezoffii*, *Cinnamomum cassia*, *Lycopodium clavatum*, Olibanum	Eliminating dampness and dredging channels	Dispelling wind and dehumidification, activating blood and removing stasis, clearing collaterals, and relieving pain
Compound Huoxue Huayu Spraying-film (Lao et al., 2018)	*Angelica*, *Carthamus tinctorius* L.	Promoting blood circulation to remove blood stasis	Angelica indications invigorate blood, fall and fall injury.
			Safflower has the effect of promoting blood circulation, dispersing blood stasis, and relieving pain
Ganoderma Triterpene Nanogel (Ye et al., 2013)	*Ganodorma lucidum*	Resistance to frostbite	Triterpenoids extracted from Ganoderma lucidum have an anti-frostbite effect
Compound Liquorice Microemulsion Gel (Wang, et al., 2020)	*Glycyrrhiza*, Sophora alopecuroide	Treatment of eczema	Clear heat and detoxify, dehumidify and relieve itching
Germinal Coating Agent (Zhao et al., 2021)	*Polygonum multiflorum, Rehmannia glutinosa, Angelica*	Treatment of loose hair, sparse or total shedding, alopecia areata, alopecia totalis, and alopecia universalis	*Polygonum multiflorum* and *Rehmannia glutinosa* can nourish liver and kidney, nourish blood and grow hair

The above is an overview of the current stage of the clinical application of common TCM films. However, in recent years, studies have also shown that the combination of TCM film preparations with physical transdermal technology including iontophoresis and ultrasound introduction can expand the scope of transdermal drug delivery (Li et al., 2022). For example, TCM acupoint iontophoresis therapy is a new and improved treatment method, which mainly uses direct current to introduce TCM ions into the diseased part through acupoints for treatment. Xie et al. found that acupoint sticking and iontophoresis combined with oral administration of TCM are significantly more effective than oral traditional Chinese medicines alone (Xie et al., 2021). In the same way, ultrasound introduction promotes drug absorption through ultrasound technology. Fan et al. reported that the effect of ultrasonic introduction of compound *Panax notoginseng* pain-relieving ointment in the treatment of rabbit knee osteoarthritis was significantly better than the control group (Fan et al., 2017). In addition to TCM films, some newly discovered medicinal film composition materials and film-forming technologies have more unique advantages and stronger therapeutic effects in clinical applications. For example, in clinical medicine, the culture of human stem cells and tumor cells plays a crucial role. The traditional cell culture method is monolayer cell culture, but it has great limitations. The hyaluronic acid-modified chitosan, graphene and other materials were used to make biofilms by means of photolithography and stamping, then construct a spheroid cell culture system. It effectively enhances the stemness and proliferation ability of some cells *in vitro*, and ensures the quality and quantity of cell culture (Ryu et al., 2019). Furthermore, the COVID-19 vaccine is the most effective means of preventing COVID-19. The newly developed vaccine-loaded nano or microparticle films can be administered orally or sublingually. The film induces protective immunity at mucosal sites, including mucosal immunity and systemic immunity. And under a large-scale epidemic, it has higher acceptability and safety. Compared with vaccine intramuscular injection, it has greater advantages (Shah et al., 2021). Moreover, conductive biomaterials based on conductive polymers, carbon nanomaterials, or conductive inorganic nanomaterials demonstrate great potential in wound healing and skin tissue engineering, owing to the similar conductivity to human skin, good antioxidant and electrically controlled drug delivery, and photothermal effect. So, infiltrating it into dressings such as films and hydrogels supplemented by electrotherapy to generate electrical stimulation can effectively promote the healing process of acute and chronic wounds at all stages (Yu et al., 2021). Besides, many 2D nanomaterials such as graphene, layered double hydroxides, etc., have been used for synaptic modulation, neuroinflammation reduction, stem cell fate modulation, and damaged nerve cell/tissue repair. Break through the difficult problems of nervous system diseases that are difficult to solve by conventional TCM films (He et al., 2022). It is expected that the above new progress in the clinical application of medicinal films can provide new inspiration and direction for the development of TCM films in the future, so that the TCM films will show greater clinical value and charm.

## 6 Perspectives

Film preparation is a leap from traditional drug delivery systems (tablets, powders, capsules) to new preparations of TCM preparations. Its development is rapid and related reports are increasing. With the in-depth research, the film gradually reflects the characteristics of high precision, simple process, exact curative effect, convenient portability, comfortable use and low cost. In addition, as a new type of controlled drug delivery system, film formulations have the advantages of stable administration, maintenance of local mucosal and plasma drug concentrations, greatly improved drug utilization, less side effects, direct contact with wounds for protection and isolation. Therefore, it has an irreplaceable role in clinical treatment, and it also conforms to the trend of modernization and reform of TCM preparations, and has a lot of room for development.

At the same time, the development of film agents also faces many challenges. In the early stage of preparation, the extraction process of Chinese herbal medicines is rough, the quality of the extracted active ingredients of Chinese herbal medicines is difficult to control, and there is no standardized, easy to quantify and visualized standards for reference. In the process of preparation, factors such as operating equipment, preparation method, sample adding sequence, dose difference, and film-forming environment will affect the character and texture of film agent to a certain extent. After the preparation is completed, the application and evaluation of the finished product also needs to be updated, including the drug content, drug content uniformity, thickness, disintegration and dissolution rate of the film formulations, etc. So It is necessary to improve or invent faster, better and more economical testing methods. In addition, the pharmacological mechanism of some TCM films to exert their efficacy is still unclear. It is still necessary to continuously study and explore its pharmacological mechanism to provide a scientific basis for the cure of certain diseases and a more assured guarantee for the use of patients.

The above-mentioned problems restrict the further development of film formulations. Therefore, this paper aims to summarize the application status of Chinese medicinal materials in the film drug delivery system, in order to provide help for the research of Chinese medicinal film for the majority of scholars. In addition, in the development direction of future drug delivery systems, some scholars have continuously proposed the use of advanced nanoscale materials as drug targeted delivery carriers. The advantage of using nanomaterials is that the drug carrier is shifted from macroscopic research to microscopic, forming intelligent targeted delivery, and accurately predicting the drug delivery rate. But whether its safety and cost-effectiveness can generalize its application remains to be further investigated. It is believed that with the continuous deepening of research, more effective and more mature TCM film production technology will be launched in the future. And the TCM film with better performance and curative effect can be produced, which can be used in clinical practice to solve more medical problems.
